# Organism-specific depletion of highly abundant RNA species from bacterial total RNA

**DOI:** 10.1099/acmi.0.000159

**Published:** 2020-09-09

**Authors:** Florian Engelhardt, Jürgen Tomasch, Susanne Häussler

**Affiliations:** ^1^​ Helmholtz Centre for Infection Research, Braunschweig, Germany; ^2^​ Institute for Molecular Bacteriology, TWINCORE, Centre for Experimental and Clinical Infection Research, Hannover, Germany; ^3^​ Department of Clinical Microbiology, Copenhagen University Hospital – Rigshospitalet, Copenhagen, Denmark; ^4^​ Cluster of Excellence RESIST (EXC 2155), Hannover Medical School, Hannover, Germany

**Keywords:** mRNA enrichment, RNA-seq, RNase H, tmRNA, *Pseudomonas aeruginosa*, rRNA depletion

## Abstract

High-throughput sequencing has become a standard tool for transcriptome analysis. The depletion of overrepresented RNA species from sequencing libraries plays a key role in establishing potent and cost-efficient RNA-seq routines. Commercially available kits are known to obtain good results for the reduction of ribosomal RNA (rRNA). However, we found that the transfer-messenger RNA (tmRNA) was frequently highly abundant in rRNA-depleted samples of *Pseudomonas aeruginosa *, consuming up to 25 % of the obtained reads. The tmRNA fraction was particularly high in samples taken from stationary cultures. This suggests that overrepresentation of this RNA species reduces the mRNA fraction when cells are grown under challenging conditions. Here, we present an RNase-H-based depletion protocol that targets the tmRNA in addition to ribosomal RNAs. We were able to increase the mRNA fraction to 93–99% and therefore outperform not only the commercially Ribo-off kit (Vazyme) operating by the same principle but also the formerly widely used Ribo-Zero kit (Illumina). Maximizing the read share of scientifically interesting RNA species enhances the discriminatory potential of next-generation RNA-seq experiments and, therefore, can contribute to a better understanding of the transcriptomic landscape of bacterial pathogens and their used mechanisms in host infection.

## Introduction

During the last decade, RNA sequencing (RNA-Seq) has increasingly become the method of choice in transcriptome studies. It replaced conventional methods such as microarrays to quantify gene-expression levels more accurately in both eukaryotic and prokaryotic organisms. RNA-seq has benefitted from decreasing sequencing costs [1]. However, also efficient depletion of the rRNA fraction and other overrepresented RNA species prior to performing an mRNA library preparation reduces overall costs and maximizes the information output on gene-expression levels.

In eukaryotes, polyadenylation of the mature mRNA facilitates mRNA enrichment, e.g. by the use of bead pulldown approaches [2]. In bacteria, depletion of highly abundant RNA is more challenging. The two most commonly used methods for the removal of non-mRNA involve a hybridization step: DNA probes with complementary sequences bind to the target RNA for subsequent removal of the hybrid. Those DNA–RNA hybrids can be captured via binding of the hybridized probes to magnetic beads, which is often accomplished by a biotin-streptavidin interaction [3] or antibody-mediated binding [4]. An alternative for DNA–RNA hybrid removal is their enzymatic digestion. For example, RNase H cleaves the RNA strand of DNA–RNA hybrids. There are also protocols involving enzymatic hybrid degradation at the cDNA stage by a duplex-specific nuclease (DSN) [5, 6].

Commercially available kits, e.g. Ribo-Zero (Illumina), which capture hybridized probes by the use of magnetic beads, are known to efficiently reduce the rRNA fraction down below 1 % in a wide range of bacterial species [7]. However, other non-mRNA fractions are reduced less efficiently. For example, when applying the protocols to total RNA of *Pseudomonas aeruginosa* up to 25 % of the reads can map to tmRNA as found in transcriptome data recorded earlier by our group and in other studies (Fig. S1, available in the online version of this article) [7, 8]. First identified in *Escherichia coli* [9], this small stable tmRNA with an average length of ca. 350 bp plays a key role in recycling of stalled ribosomes and tagging of unfinished peptides for degradation in bacteria [10]. Another commercially available kit, the Ribo-off kit (Vazyme), is based on the depletion of DNA–RNA hybrids via RNase-H digestion. The rRNA targets are very efficiently depleted in human samples [11] and a broad range of bacteria [2].

Here we describe an RNase-H-digestion-based, organism-specific and easily customizable method for removal of overrepresented RNA species that goes beyond rRNA removal. We designed *P. aeruginosa *-specific DNA probes, which allowed the efficient removal of tmRNA fragments in addition to the depletion of *P. aeruginosa* 16S and 23S rRNA. Our protocol outperformed the commercially available Ribo-Zero (Illumina) and Ribo-off kit (Vazyme) with a significant increase in the fraction of *P. aeruginosa* mRNA-mapped reads to more than 95 % of the overall mapped reads.

## Methods

### Used bacterial strain and culture conditions

Planktonic cultures were inoculated with single colonies of *P. aeruginosa* strain PA14 and incubated overnight in lysogenic broth (LB) at 37 °C while shaking at 180 r.p.m. Subsequent main cultures were inoculated at an OD_600_ of 0.05 in LB and grown at 37 °C while shaking at 180 r.p.m. until they reached OD_600_=2.0±0.1 (late exponential phase) or for 12 h (stationary phase).

### Cell harvest and extraction of total RNA

Overall, 1 ml of the main culture was mixed with 1 ml RNAprotect Cell Reagent (ID 76526, Qiagen) and incubated at room temperature (RT) for 10 min. After a centrifugation at 8000 r.p.m. at RT for 15 min, the supernatant was discarded and samples were stored at −80 °C until use.

For cell lysis, thawed samples were centrifuged for 5 min at 8000 r.p.m. and the supernatant was removed. Then, 100 µl of TE buffer containing 800 µg ml^−1^ lysozyme were added to the cells and mixed thoroughly by vortexing for 30 s. After an incubation for 10 min at RT, intermitted by occasional short vortexing, 350 µl of RLT buffer (from RNeasy Mini Kit; ID 74106, Qiagen) containing 1 % mercaptoethanol were added to the sample. The mixture was inverted several times and incubated for 1 h at −80 °C. Samples were then thawed on ice and loaded into a QIAshredder column (ID 79656, Qiagen), followed by centrifugation for 2 min at 14 000 r.p.m. RNA was extracted from the supernatant with the RNeasy Mini Kit (ID 74106, Qiagen) according to the manufacturer’s instructions. Subsequently, remaining DNA was removed from the extracted RNA samples by treatment with the DNA-free DNA Removal kit (ID AM1906, Invitrogen) according to the manufacturer’s protocol.

### Design of organism-specific probes for *P. aeruginosa*

Sequences of 16S rRNA (NC_002516.2:c5269259-5267724), 23S rRNA (NC_002516.2:c6042736-6039846) and tmRNA (NC_002516.2:c901520-901872) genes of *P. aeruginosa* PAO1 were downloaded from pseudomonas.com (Tables S1 and S2). Sequence differences between the rRNA genes of the *P. aeruginosa* reference strain PAO1 and PA14 were checked and considered negligible.

The reverse complement sequences of the respective gene sequences were segmented into fragments with a size of 50±5 bp. DNA nucleotides were designed to exhibit similar binding affinities to the target RNA, so that they exhibited homogenous melting temperatures (calculated after the Marmur Doty formula [12]) rather than to express a uniform length (see Table S3 for used R script). In total, 94 probe sequences for the 16S rRNA, 23S rRNA and the tmRNA gene were obtained.

Check for off-target hybridization was carried out by blast analysis [13] and showed no cross-hybridization within the genome or the probe pool. Unmodified DNA oligonucleotides (Table S4) were used in an equimolar mixed probe pool within the RNA depletion experiment.

### Depletion of overrepresented RNAs from total RNA

The protocol for hybridization of the designed oligonucleotides to their target RNAs and the subsequent cleavage of the hybridized RNA by the RNase H was adapted from Adiconis *et al.* [11]. In brief, hybridization was carried out by mixing 1 µg total RNA with 1 µg of the equimolar probe pool in 1 µl 5× hybridization buffer (500 mM Tris-HCl and 1 M NaCl) and 2 µl nuclease-free water. The hybridization mix was incubated for 2 min at 95 °C. To facilitate the annealing of the designed DNA oligonucleotides to the target RNA, the temperature was slowly reduced by −0.1 °C per second to 45 °C. Altogether, 10 U of the Hybridase Thermostable RNase H (ID H39500, Lucigen) and 2 µl 5× reaction buffer (250 mM Tris-HCl, 500 mM NaCl and 100 mM MgCl_2_) were added and the digestion was performed in a final volume of 10 µl for 30 min at 37 °C. RNAClean XP magnetic beads (ID A66514, Beckman Coulter) were used in a 1 : 1 ratio to purify the RNA and 15 µl of the eluate were incubated with 4 U rDNase I in 1× DNAse I buffer from the DNA-free kit (ID AM1906, Invitrogen) with a final volume of 20 µl for 30 min at 37 °C. DNase reaction mix was purified with 1× RNAClean XP magnetic beads obtaining 17 µl eluate. Fragment size distribution was assessed by the use of the Bioanalyzer (2100 Bioanalyzer Instrument, Agilent) and RNA concentration was determined by photometric measurement (NanoDrop ND-1000, Thermo Scientific).

### Ribo-off as reference kit for depletion efficiency

For the comparison of the depletion efficiency of the mRNA enrichment protocol described above, the commercially available Ribo-off rRNA Depletion Kit (Bacteria) (ID N407, Vazyme) was used. Total RNA samples were treated for rRNA depletion as indicated by the manufacturer’s instructions.

### Preparation of the sequencing library and Illumina NovaSeq sequencing

Sequencing libraries of the depleted RNA samples were prepared with the NEBNext Ultra II Directional RNA Library Prep Kit (ID E7760L, New England Biolabs) accordingly to the manufacturer’s instructions and then sequenced with an Illumina NovaSeq 6000 Sequencing System (50 bp paired-end reads, S1 flow cell).

### Quality assessment and analysis of the sequencing data

A first evaluation of the sequencing data quality was performed by using FastQC (version 0.11.4, https://github.com/s-andrews/FastQC). For removal of potentially remaining adapter reads and clipping of the reads, Fastq-Mcf (version 1.04.803, https://github.com/ExpressionAnalysis/ea-utils/blob/wiki/FastqMcf.md) was used. Mapping was carried out by bowtie2 [14] (version 2.2.6, https://github.com/BenLangmead/bowtie2) based on genome data retrieved from NCBI (CP000438.1) and featureCounts [15] (version 1.6.4, http://subread.sourceforge.net/) was used to determine the number of sequencing reads that can be assigned to each genomic feature. The calculation of the read fractions for the different RNA species, the correlation analysis and the generation of the corresponding figures were performed by a custom R script.

## Results

### Depletion of highly abundant RNA species with an organism-specific probe design

**Fig. 1.**
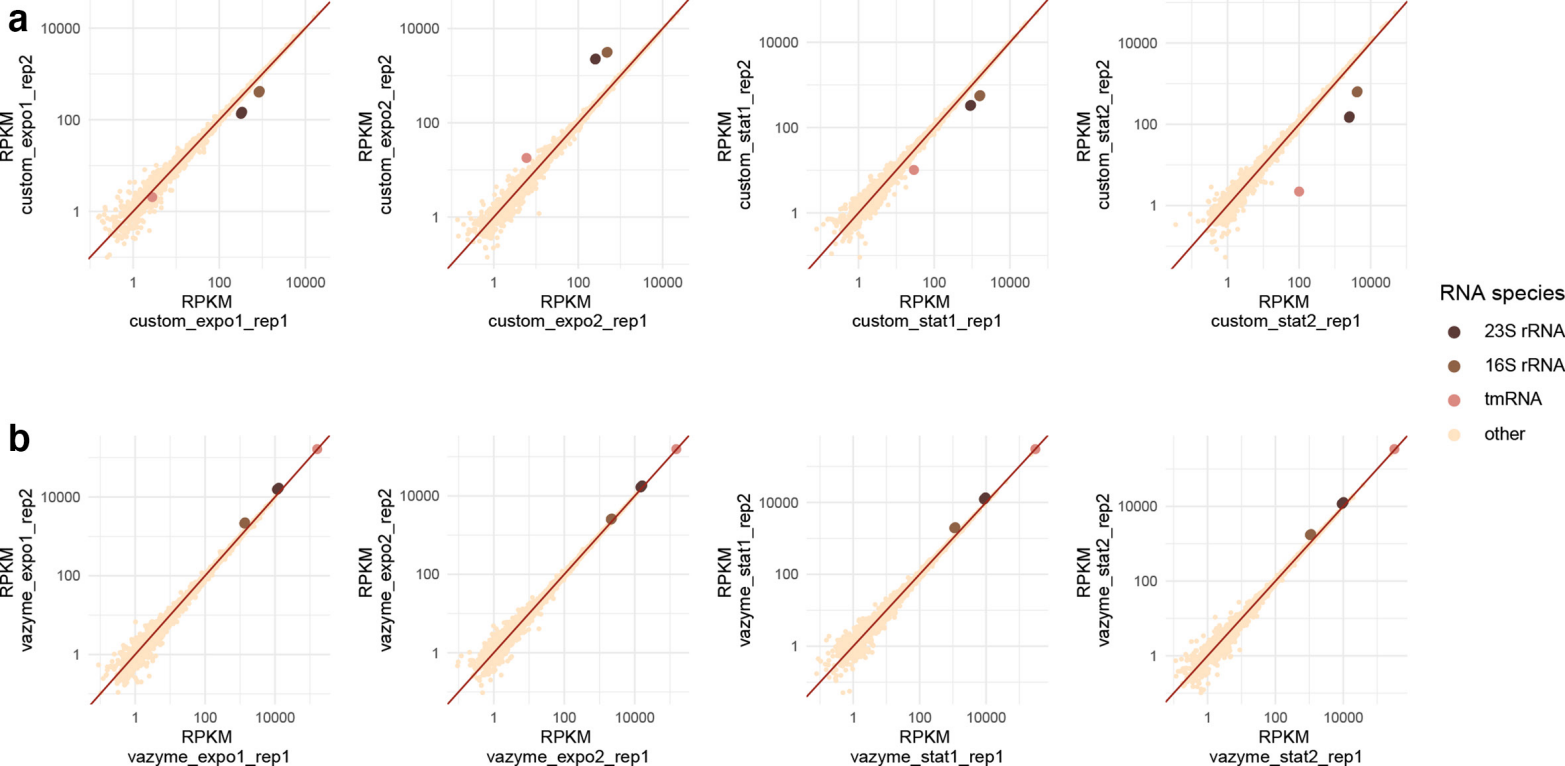
Correlation analysis of expression profiles between the technical replicates. The reads per kilo base per million mapped reads (RPKM) of the replicates treated with (a) the custom depletion method or (b) the Ribo-off kit were used for correlation analysis; custom, custom depletion technique; vazyme, Ribo-off (Vazyme).

We compared the efficiency of our custom-made *P. aeruginosa* targeted depletion of non-mRNA to that of the commercial Ribo-off kit (Vazyme), both of which are based on an RNase-H digestion of RNA–DNA hybrids. Therefore, total RNA from *P. aeruginosa* cells harvested in the exponential and stationary growth phase was prepared in two biological replicates. The two independent depletion methods were applied on the same RNA samples in two technical replicates. Both protocols were stable and high correlation of gene-expression profiles across biological and technical replicates were achieved (Figs 1 and S1, Table S5 for the number of total reads per sample).

**Fig. 2.**
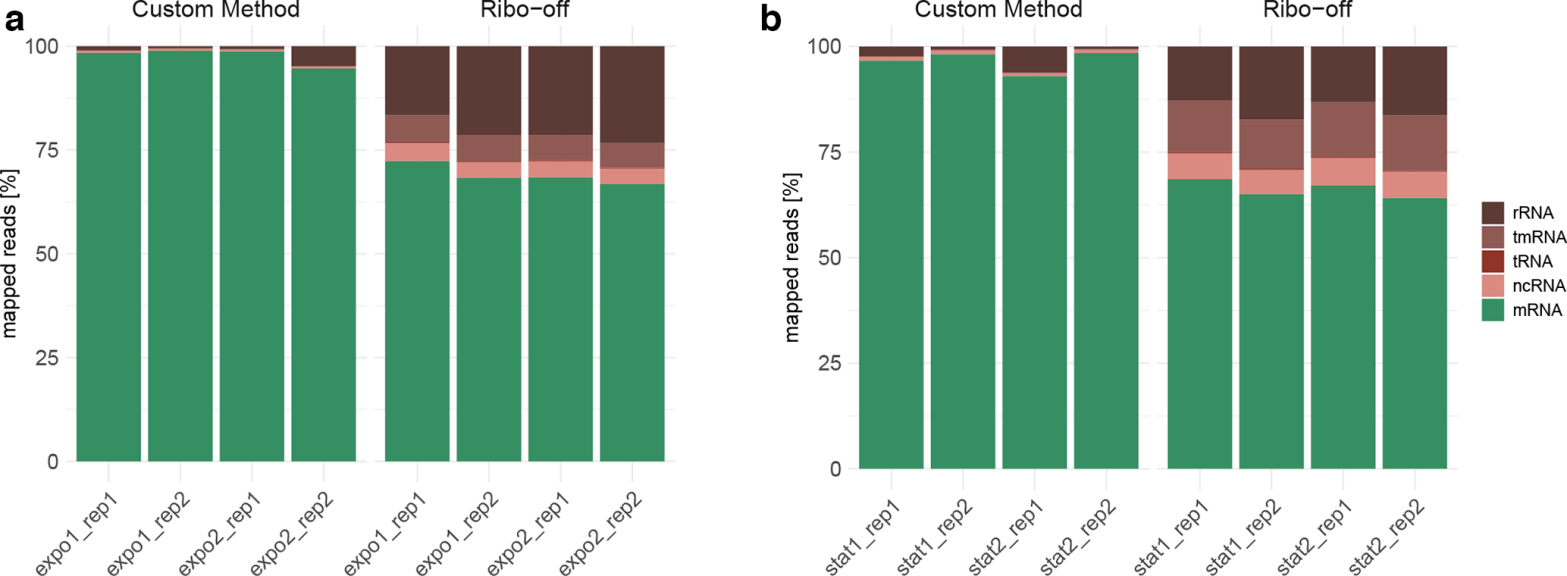
Percentage of the various fractions of RNAs among the entirety of mapped reads. Total RNA was prepared from (a) exponential-phase and (b) stationary-phase cultures and the custom-made (left) and the Ribo-off (right) depletion protocols were applied. The four columns represent two biological replicates, with two technical replicates each. Absolute read count values are stated in Table S5.

We found that the specific targeting led to a more effective depletion of the *P. aeruginosa* rRNA in our custom-made protocol (Fig. 2, Table S5). The total mapped RNA contained less than 5 % rRNA in samples from cells harvested both in the exponential and stationary phase, while the relative fraction of rRNA upon application of the Ribo-off kit was as high as 17–23 %. With this, the depletion efficiency of our protocol was also higher than in previously prepared samples where the commonly used Ribo-Zero kit has been used (rRNA median fraction of 21 %, Figs S2 and S3).

In our custom-made protocol we did not only target the *P. aeruginosa* rRNA but also the tmRNA. This was highly successful, as almost no tmRNA remained after the application of our depletion protocol. In contrast, the Ribo-off-kit-treated samples contained up to 6 % tmRNA, when the total RNA was prepared from exponential-phase cultures and at least 12 % when prepared from stationary phase. Similar amounts of tmRNA have been detected in RNA samples upon application of the Ribo-Zero kit.

A maximum of 72 % of the reads from the Ribo-off-treated samples mapped to coding sequences, while the proportion of mRNA reads was at least 93 % in the samples where the RNA has been depleted with the more targeted DNA probe design established by our group.

Of note, the percentage of the non-coding RNA (ncRNA) among all mapped reads across all samples was below 1 % after following our custom-made depletion protocol. In contrast, 4–7 % ncRNAs were found in the RNA samples, in which the Ribo-off depletion protocol was applied (for a more detailed analysis of the distribution of ncRNAs in this fraction see below). Furthermore, the transfer RNA (tRNA) fraction was larger in the Ribo-off-treated samples (0.2–0.3 %) than in the samples treated with our protocol (0.09–0.12 %).

### Correlation analysis show no systematic bias in transcript levels of coding sequences

**Fig. 3.**
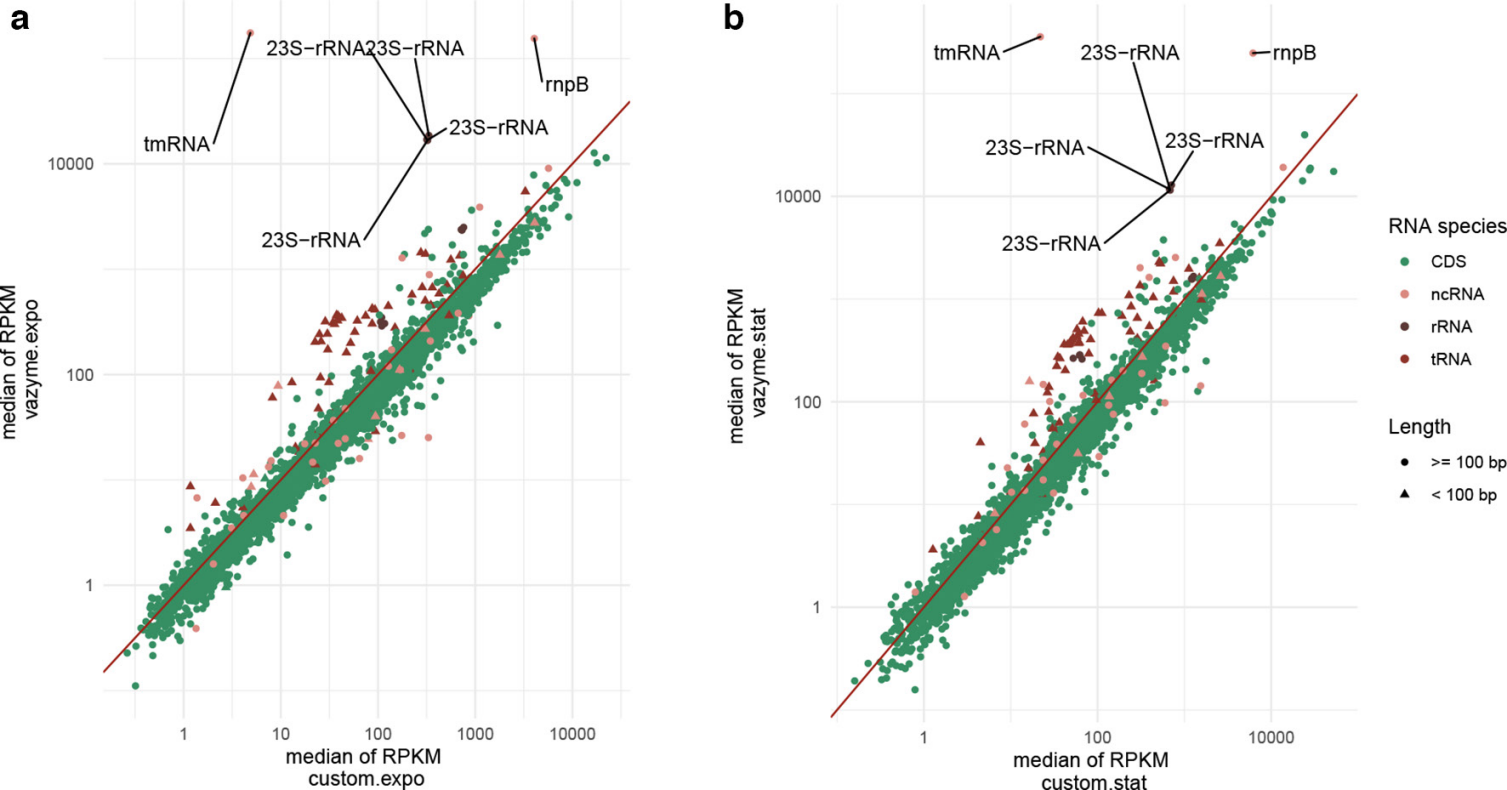
Comparison of gene-expression profiles between samples treated with the Ribo-off versus our custom-made depletion protocol. The median of the reads per kilo base per million mapped reads (RPKM) of two biological and two technical replicates from (a) exponential or (b) stationary growth phase were used for correlation analysis; gene names of strongly deviating values are indicated; custom, custom depletion technique; vazyme, Ribo-off (Vazyme); expo, exponential phase; stat, stationary phase. Correlation plots of not aggregated data are provided in Figs S4 and S5.

In order to rule out that biases in gene-expression profiles are introduced by the application of the specific depletion protocols, we determined the reads per kilo base per million mapped reads (RPKM) for each gene and compared the values of our method to those of the Ribo-off kit. No systematic divergences in the abundance of reads mapping to coding sequences (CDS) could be observed (Figs 3, S4 and S5).

As expected, the detected abundance of rRNA and tmRNA was higher in all samples treated with the Ribo-off kit. Furthermore, transcript levels of the tRNA genes were higher. As we did not explicitly target the tRNAs with our DNA probe design, the more stringent exclusion of small RNAs with an extensive secondary structure could be the reason for their lower abundance in our custom-made protocol treated samples. In addition, more *rnpB* gene transcripts encoding the ncRNA component of the ribonuclease RNase P, could be identified in the samples treated with the Ribo-off kit. Of note, these transcripts accounted to for at least 18 % of the reads mapping to ncRNA genes in the Ribo-off-treated RNA. A size-dependent depletion of this non-coding RNA in our protocol seems unlikely (*rnpB* has a size of 209 bp). Furthermore, blast analysis revealed no off-target hybridization of any of the designed probes to the rnpB RNA.

## Conclusion

We have established an efficient protocol for a highly specific depletion of non-mRNA such as 16S and 23S rRNA and tmRNA species from preparations of total *P. aeruginosa* RNA. As a result, the fraction of mRNA increased to >93 %. No overall bias was introduced and the gene-expression profile compared well to that of the mRNA-profile of the Ribo-off-treated RNA controls.

We found that depletion of RNAs with a shorter length and a more extensive secondary structure such as tRNAs and other selected ncRNAs was stronger, although those RNAs were not explicitly targeted by our established method. We assume that these RNAs were depleted during the purification steps as short length and distinct 3D structure presumably impair their binding to the surface of the magnetic beads used for purification. Given that as a consequence the relative sequence information of the mRNA fragments increase, the stronger depletion of tRNAs and *rnpB* does not lead to a drawback of the depletion protocol.

The easily expendable spectrum of target sequences allows not only to deplete any additional *P. aeruginosa* RNA but also to target non-mRNAs from other bacterial species. Thus, the newly established mRNA-enrichment protocol is particularly suited for high-throughput transcriptome analyses with RNA samples from the same species and represents a cost-efficient alternative. Its use can boost the elucidating power of studies examining the transcriptomic landscape of pathogenic bacteria and, therefore, allows deeper insights into the process of bacterial pathogenesis, e.g. in host-pathogen transcriptome experiments (Dual-Seq).

## Supplementary Data

Supplementary material 1

Supplementary material 2
