# Supplementary material for: Organism-specific depletion of highly abundant RNA species from bacterial total RNA
Source: Access Microbiol. 2020 Sep 9;2(10):acmi000159. doi: 10.1099/acmi.0.000159 (PMC7660241; doi:10.1099/acmi.0.000159)
Supplement: Supplementary material 1 [file acmi-2-159-s001.pdf]

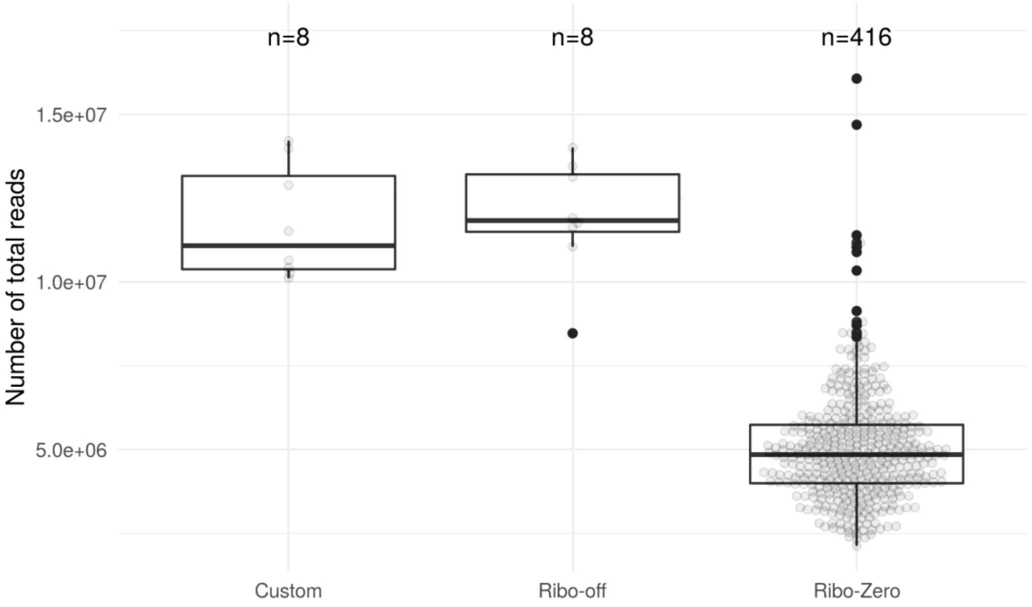

Supplementary Figure S1: Library size of RNA samples treated with different depletion methods. Total number of obtained sequencing reads per sample. RNA samples depleted with Ribo-Zero™ Magnetic Kit (Bacteria) (ID RZMB12324 and MRZ11124C, Illumina) were isolated from 414 clinical isolates of *Pseudomonas aeruginosa* as well as PA14 and PAO1 and corresponding data was published earlier by Hornischer *et al.* [8] (available at Gene Expression Omnibus, GSE123544). Horizontal lines in boxplots represent the median; Custom, custom depletion technique; Ribo-off, Ribo-off (Vazyme); Ribo-Zero, Ribo-Zero™ Magnetic Kit (Bacteria) (Illumina).

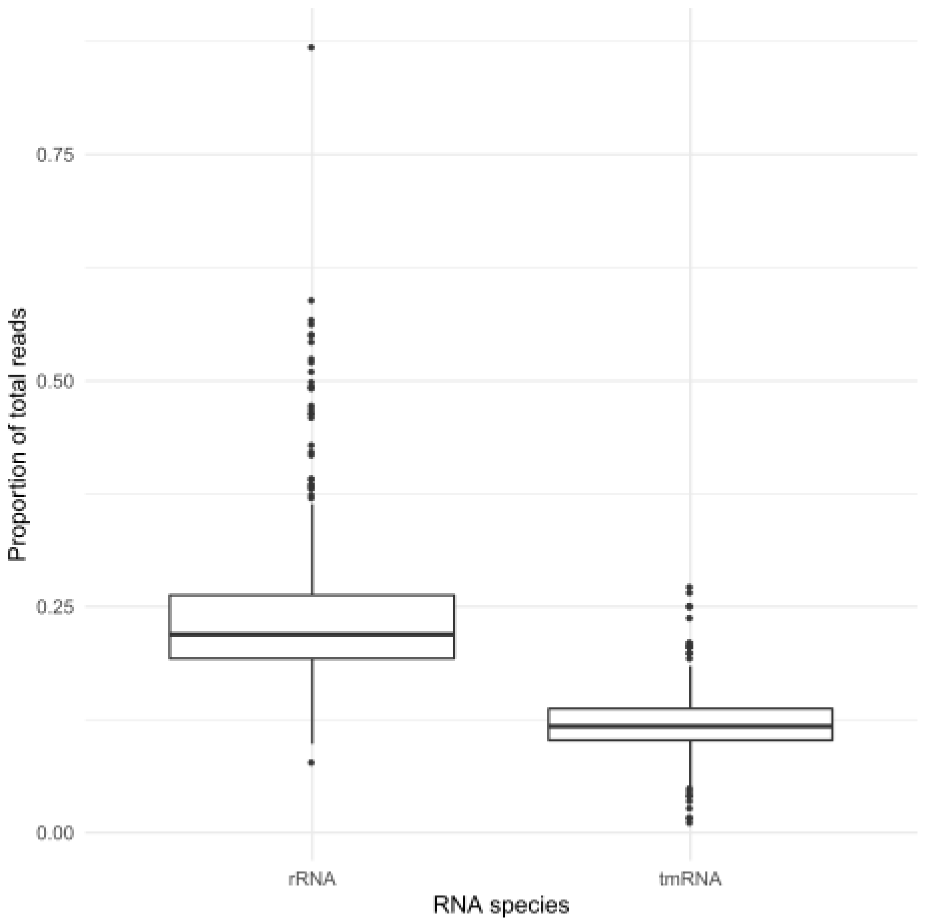

Supplementary Figure S2: rRNA and tmRNA read share of transcriptomic data obtained from RNA samples treated with Ribo-Zero™ Magnetic Kit (Bacteria) (Illumina). RNA samples were isolated from 414 clinical isolates of *Pseudomonas aeruginosa* as well as PA14 and PAO1. Data was published earlier by Hornischer *et al.* [8] (available at Gene Expression Omnibus, GSE123544). Horizontal lines in boxplots represent the median.

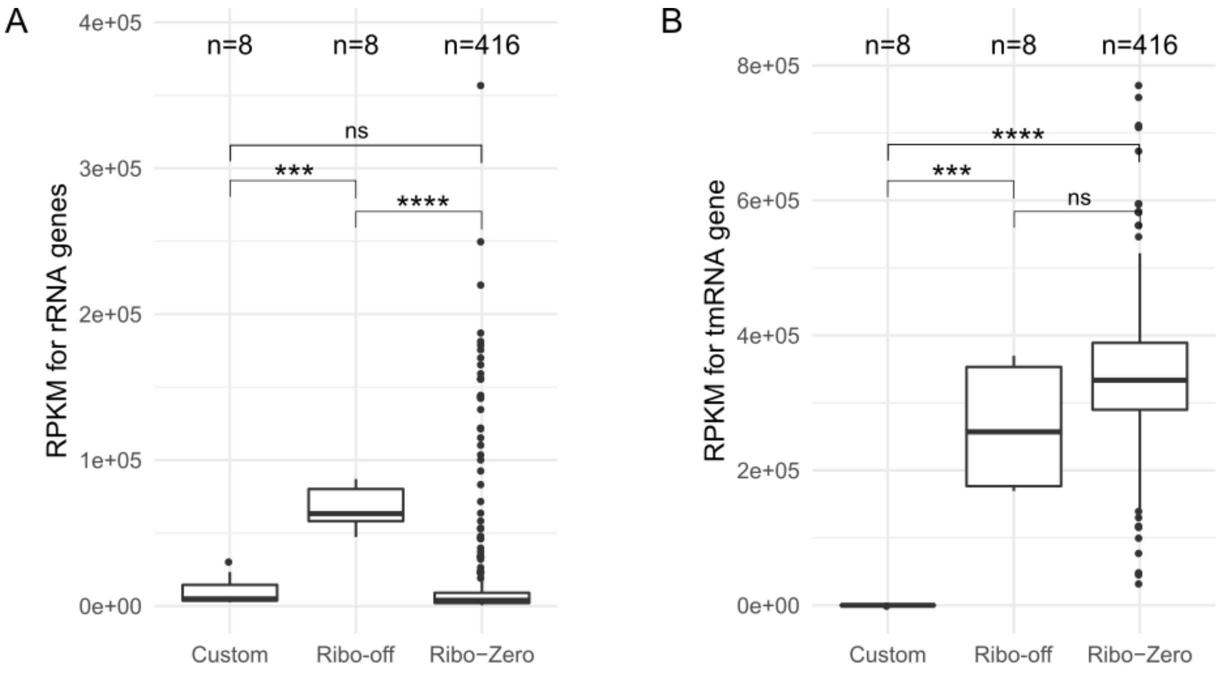

Supplementary Figure S3: Number of reads for rRNA and tmRNA genes in samples treated with different depletion methods. Depicted are the reads per kilo base per million mapped reads (RPKM) for (A) the rRNA genes or (B) the tmRNA gene. RNA samples depleted with Ribo-Zero™ Magnetic Kit (Bacteria) (ID RZMB12324 and MRZ11124C, Illumina) were isolated from 414 clinical isolates of *Pseudomonas aeruginosa* as well as PA14 and PAO1 and corresponding data was published earlier by Hornischer *et al.* [8] (available at Gene Expression Omnibus, GSE123544). Horizontal lines in boxplots represent the median. Significance was tested by application of the Mann-Whitney U test. Custom, custom depletion technique; Ribo-off, Ribo-off (Vazyme); Ribo-Zero, Ribo-Zero™ Magnetic Kit (Bacteria) (Illumina); ****, $p \leq 0.0001$; ***, $p \leq 0.001$; ns, not significant.

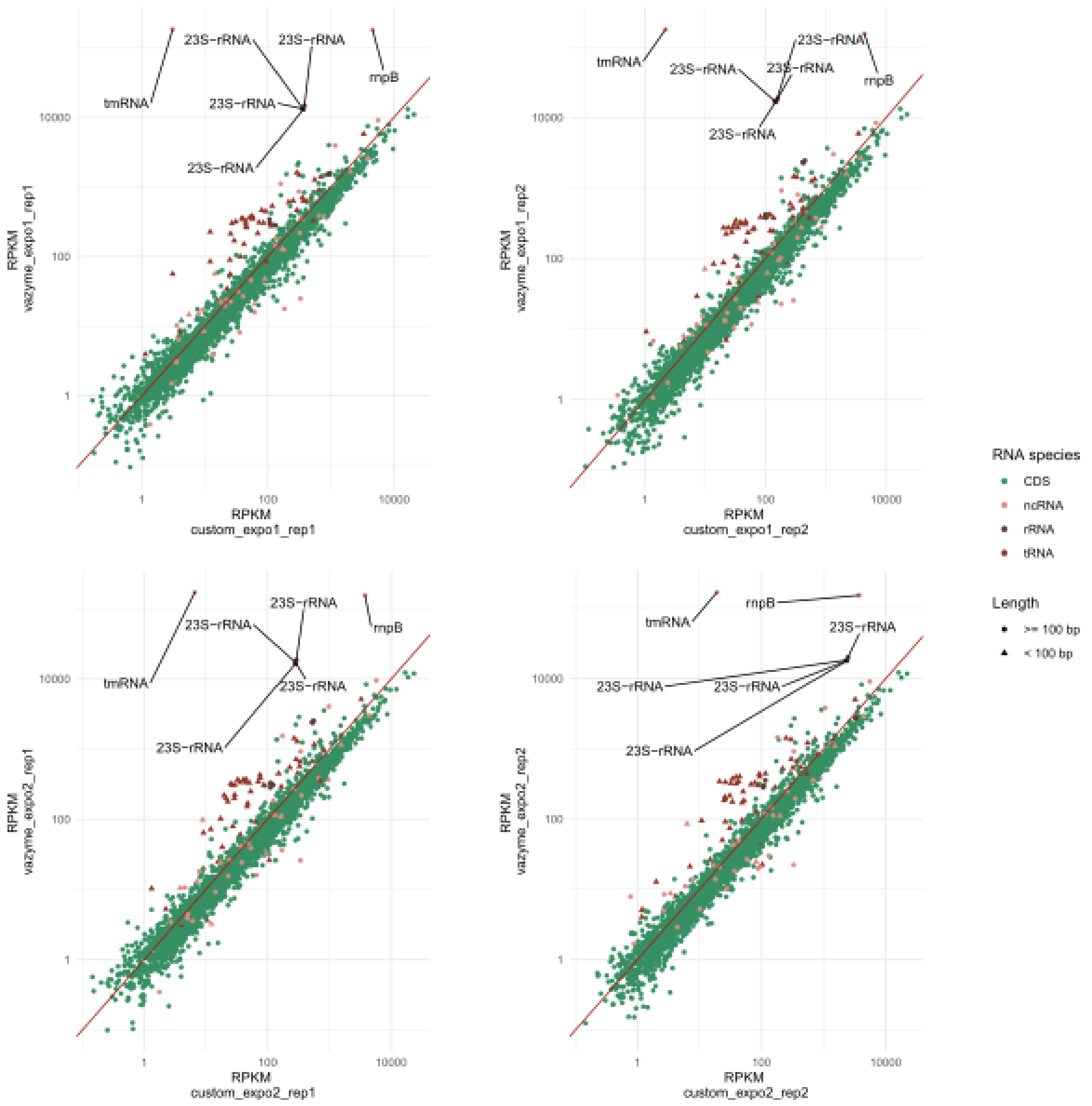

Supplementary Figure S4: Correlation analysis of expression profiles between samples treated with one of the evaluated depletion protocols during exponential growth phase. The reads per kilo base per million mapped reads (RPKM) of all biological and technical replicates taken in exponential growth phase were used for correlation analysis; gene names of strongly deviating values are indicated; custom, custom depletion technique; vazyme, Ribo-off (Vazyme).

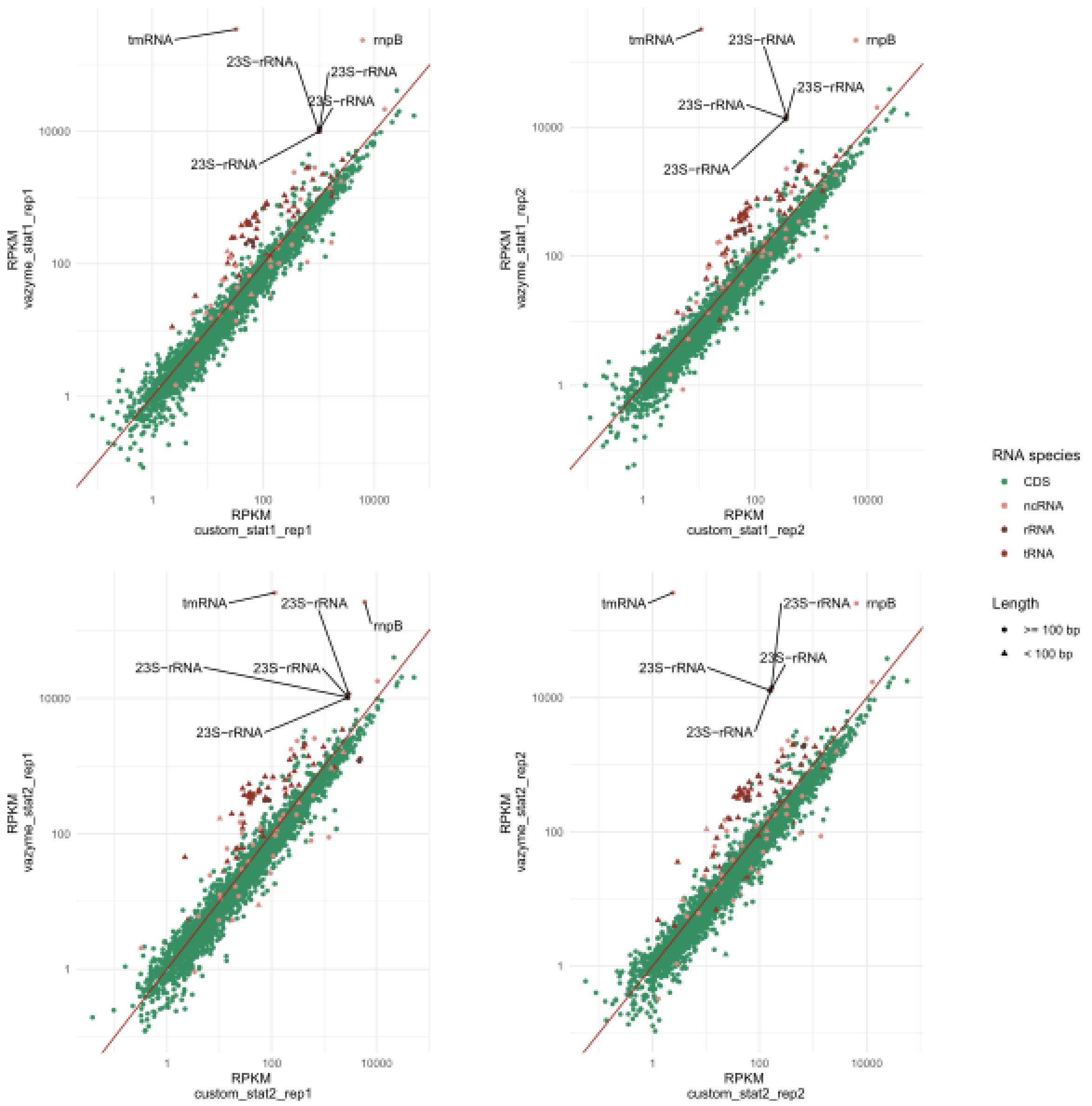

Supplementary Figure S5: Correlation analysis of expression profiles between samples treated with one of the evaluated depletion protocols during stationary growth phase. The reads per kilo base per million mapped reads (RPKM) of all biological and technical replicates taken in stationary growth phase were used for correlation analysis; gene names of strongly deviating values are indicated; custom, custom depletion technique; vazyme, Ribo-off (Vazyme).
